# Burden of Illness Associated with Peripheral and Central Neuropathic Pain among Adults Seeking Treatment in the United States: A Patient-Centered Evaluation

**DOI:** 10.1111/pme.12502

**Published:** 2014-07-08

**Authors:** Caroline Schaefer, Rachael Mann, Alesia Sadosky, Shoshana Daniel, Bruce Parsons, Edward Nieshoff, Michael Tuchman, Srinivas Nalamachu, Alan Anschel, Brett R Stacey

**Affiliations:** * Covance Market Access Services Inc.Gaithersburg, Maryland, USA; † Covance Market Access Services Inc.San Diego, California, USA; ‡ Pfizer, Inc.New York, New York, USA; § Covance Market Access Services Inc.Conshohocken, Pennsylvania, USA; ¶ Rehabilitation Institute of Michigan/Wayne State UniversityDetroit, Michigan, USA; ** Palm Beach Neurological CenterPalm Beach Gardens, Florida, USA; †† International Clinical Research InstituteOverland Park, Kansas, USA; ‡‡ Rehabilitation Institute of ChicagoChicago, Illinois, USA; §§ Oregon Health & Science UniversityPortland, Oregon, USA

**Keywords:** Neuropathic Pain, Pain Assessment, Health-Related Quality of Life, Health Status, Burden of Illness, Patient-Reported Outcomes

## Abstract

**Objective:**

The aim of this study was to evaluate patient-reported burden associated with peripheral and central neuropathic pain (NeP) by pain severity and NeP condition.

**Design:**

Six hundred twenty-four subjects with one of six NeP conditions were recruited during routine office visits. Subjects consented to retrospective chart review and completed a one-time questionnaire (including EuroQol-5 dimensions, 12-item Short-Form Health Survey, Brief Pain Inventory-Short Form, Medical Outcomes Study Sleep Scale, Hospital Anxiety and Depression Scale, and demographic and clinical characteristics). Pain severity scores were used to stratify subjects by mild, moderate, and severe pain. Summary statistics and frequency distributions were calculated. Differences by severity level were compared using Kruskal–Wallis (continuous variables) and chi-square or Fisher's exact test (categorical variables). Effect size was computed with Cohen's *d* (mild vs severe).

**Results:**

Subjects' mean age was 55.5. The majority (80.8%) had moderate or severe pain. Patient-reported outcomes (health status, physical and mental health, pain interference with function, sleep, anxiety, and depression) were significantly worse among subjects with greater pain severity (all *P* < 0.0001). Severe pain subjects were negatively impacted by ≥30% in each outcome compared with mild pain subjects; standardized effect size was moderate for anxiety (0.59) and large (>0.95) for all others. The observed burden was most substantial among chronic low back pain-NeP, although the pattern of disease burden was similar across the six NeP conditions.

**Conclusions:**

Subjects across NeP conditions exhibited high pain levels, which were significantly associated with poor function, compromised health status and sleep, and increased anxiety and depression. Results indicate substantial patient burden across broad NeP, particularly among subjects with severe pain.

## Introduction

Neuropathic pain (NeP), defined as “pain arising as a direct consequence of a lesion or disease affecting the somatosensory system,” is a *sequela* of a diverse set of diseases and medical conditions and can be classified as peripheral or central, depending on the origin in the nervous system [1],[2]. Peripheral neuropathic conditions include painful diabetic peripheral neuropathy (pDPN), post-herpetic neuralgia, chemotherapy-induced peripheral neuropathy, human immunodeficiency virus (HIV) sensory neuropathy, post-traumatic/post-surgical (PTPS) NeP, chronic low back pain (CLBP) with NeP, small fiber neuropathy, and trigeminal neuralgia. Central neuropathic conditions include spinal cord injury (SCI)-related NeP, multiple sclerosis-related NeP, and post-stroke NeP [3].

One study estimated that the prevalence of all types of NeP among adults in the United States ranges from 3% to 12%, depending on criteria used to determine prevalence [4]. Additionally, the prevalence of NeP varies widely by underlying NeP condition [5],[6]. For example, in the United States, the estimated prevalence of CLBP-associated NeP is 2,100 per 100,000, whereas the estimated prevalence is 600 per 100,000 for pDPN, 20 per 100,000 for SCI-related NeP, and 15 per 100,000 for HIV sensory neuropathy [6]. It is generally believed that the prevalence of NeP will continue to increase due to the aging of the population and higher survival rates from conditions that are associated with NeP (such as cancer, HIV infection, and diabetes) [7].

NeP is chronic, potentially debilitating, and results in an incremental burden to patients beyond that of the underlying condition. Much of the research on the patient burden of NeP has focused on common peripheral NeP conditions, such as pDPN, PTPS NeP, and CLBP-NeP 8–20. No published studies were identified that comprehensively assessed the burden of illness from the patient's perspective (including pain severity, health status, function, sleep, anxiety, and depression) in the United States across a broad range of peripheral and central NeP conditions. In Europe, studies have been conducted among broader samples of NeP, although the extent to which specific NeP conditions were represented in these studies was not always reported and more specific measures of disease burden, such as impact on sleep, mood, and function, were not consistently included 7,21–26. Physical and mental health status, based on the 12-item Short-Form Health Survey (SF-12), in two studies conducted among broader NeP samples were similar to that observed in studies of pDPN [11],[14],[23],[24]. Studies of specific NeP conditions and broader NeP samples alike found greater patient burden among those with more severe pain [7],[11],[14]. A more thorough understanding of the impact of NeP from the patient's perspective across a broader range of NeP conditions would be beneficial and may contribute to more informed health care decisions.

The objectives of this research, therefore, were to characterize the patient-reported burden associated with peripheral and central NeP in routine clinical practice in the United States with respect to sociodemographic and clinical characteristics, health status, physical and mental health, pain interference with function, sleep, anxiety, and depression, and to evaluate any differences by pain severity and NeP condition.

## Methods

### Study Design

This cross-sectional, observational study recruited a convenience sample of subjects with 1 of 6 NeP conditions of interest between September 2011 and June 2012 from 33 community-based physician practices across the United States, including 9 general practitioners (GPs), 7 neurologists, 6 pain specialists, 3 endocrinologists, as well as 8 other specialists (e.g., orthopedist, infectious disease specialist, podiatrist, rheumatologist, etc). Given the objective to observe characteristics of NeP subjects in routine clinical practice, a brief feasibility survey was sent to 711 GPs and specialists treating NeP patients. This survey described the study design and inclusion/exclusion criteria, and included questions about site characteristics, such as institutional review board (IRB) requirements, previous research experience, and NeP patient volume. A total of 210 sites responded and 149 expressed initial interest in participating. Based on responses to the feasibility survey, 44 sites were prioritized for further evaluation to determine their ability to identify subjects and participate in the study; among eligible sites, priority was given to those with the highest number of potential subjects. Selected sites received training on the protocol, including the study inclusion and exclusion criteria, prior to study initiation. This study was approved by a central IRB, Concordia Clinical Research (Cedar Knolls, NJ, USA).

Collectively, selected sites were asked to identify eligible subjects as they presented for routine office visits. Adult subjects (≥18 years) diagnosed with 1 of 6 target NeP conditions at least 6 months ago who also were managed at the physician's practice for at least 6 months were eligible for the study. Subjects also were required to read and understand English, and must have experienced symptoms due to neuropathy for at least 3 months prior to the study. Finally, subjects were required to be willing and able to provide written informed consent, including consent for site study staff to obtain information from the subject's medical chart. Subjects were not eligible for the study if they had participated in an investigational drug study 6 months prior; had a serious or unstable medical or psychological condition that, in the opinion of the physician, would compromise participation in the study; or had a concomitant illness unrelated to NeP that may confound the assessment of NeP (e.g., fracture, lupus, rheumatoid arthritis). Based on site enrollment logs, approximately 45% of NeP patients who presented for office visits at study sites during the study enrollment period were formally screened for enrollment; patients known by the sites to be ineligible (e.g., who did not have one of the NeP types of interest) were not formally screened. A total of 624 subjects with NeP were enrolled in the study from 637 who were formally screened. Data are not available for the 13 potential subjects who failed screening.

### Data Collection

Subjects were asked to complete a self-administered one-time questionnaire. The questionnaire assessed demographics and clinical characteristics and included the following validated measures: the Brief Pain Inventory-Short Form (BPI-SF) [27], the 12-item Short-Form Health Survey, version 2, 1-week recall (SF-12v2) [28], the EuroQol 5-dimensions (EQ-5D) [29], the Medical Outcomes Study Sleep Scale (MOS-SS) [30], and the Hospital Anxiety and Depression Scale (HADS) [31],[32].

The BPI-SF includes 4 items measuring pain severity (worst, least, average, current), whose mean comprises the Pain Severity Index, and 7 items measuring pain interference with function, whose mean comprises the Pain Interference Index [27]. Items were assessed on 11-point numeric rating scales ranging from 0 (no pain) to 10 (pain as bad as you can imagine).

The SF-12v2 contains 12 items assessing 8 domains of health status. Composite physical component summary (PCS) and mental component summary (MCS) scores were calculated. Scores range from 0 to 100 with higher scores indicating better health status [28],[33].

The EQ-5D is a 5-item general health status and utility measure [29]. Health state valuation scores range from −0.11 to 1.00 with higher scores indicating better health status.

The MOS-SS includes 12 items assessing sleep, with 9 of the 12 items comprising the Sleep Problems Index, ranging from 0 to 100, with higher scores indicating worse sleep [30]. For 2 domains (sleep adequacy and quantity), higher scores indicate better sleep.

The HADS is a 14-item self-reporting tool (7 items each for anxiety and depression). Scores range from 0 to 21, with higher scores representing poorer emotional well-being. Scores of 0 to 7 represent “normal,” 8 to 10 “mild,” 11 to 14 “moderate,” and 15 to 21 “severe” levels of anxiety and depression [31],[32].

In addition, the participating physician or site coordinator conducted a 6-month retrospective review of the subject's medical chart to capture clinical characteristics and NeP-related medications and health care resource use.

### Statistical Analyses

Summary statistics, means and standard deviations (SDs) for continuous variables and frequency distributions for categorical variables were used to describe the sample. Summary statistics are presented for all available data, and data were consistently available for more than 97.0% of the sample. BPI-SF Pain Severity Index scores were used to classify pain severity as mild (0 to 3), moderate (4 to 6), and severe (7 to 10) [34],[35]. To evaluate the association between pain severity levels or NeP conditions and other patient-reported outcomes, the Kruskal–Wallis test was used for continuous variables; as such, while the means and SDs were presented for continuous variables for each group, the *P* value presented was based on the ranks. Chi-square or Fisher's exact tests were used to examine the association for categorical variables. Statistical significance was evaluated at the 0.05 level. Standardized effect size for patient-reported outcomes between mild and severe subjects was computed with Cohen's *d*. For Cohen's *d*, an effect size of 0.2 to 0.3 was considered a “small” effect, around 0.5 a “medium” effect, and 0.8 and higher was considered a “large” effect [36]. All analyses were performed using PC-SAS version 9.1.3 (SAS Institute, Cary, NC, USA).

## Results

### Demographic and Clinical Characteristics

The 6 NeP conditions were evenly represented among the 624 subjects, with not more than 18% of the total sample coming from any one NeP condition (Table 1). Table 2 presents the demographic and clinical characteristics of the sample overall and by pain severity, unless otherwise noted. The mean (SD) age was 55.5 (13.7) years and 346 (55.4%) were male. The majority of the sample was white (71.8%) and non-Hispanic (87.0%), although all racial and ethnic groups with the exception of native Hawaiian/other Pacific Islander were represented. Overall, the majority (59.4%) of subjects completed education beyond high school.

**Table 1:** Case definitions of NeP conditions of interest

NeP Condition	Peripheral or Central NeP	N (% of sample)	Case Definition
HIV-related peripheral NeP (HIV-NeP)	Peripheral	103 (16.5)	Subjects with HIV and neuropathies including distal symmetrical polyneuropathy, inflammatory demyelinating polyneuropathy, progressive polyradiculopathy, mononeuropathy multiplex, autonomic neuropathy, and diffuse infiltrative lymphocytosis syndrome for at least 3 months, confirmed by a neurologist, using established diagnostic criteria.
Post-trauma or post-surgical NeP (PTPS NeP)	Peripheral	100 (16.0)	Patients who experience neuropathic pain following a known injury or medical intervention. Pain symptoms may be felt at the site of the injury and/or radiate, usually away from the site in the normal distribution of the nerve involved. Pain must be present at least 3 months following the injury or intervention with characteristic NeP qualities.
SCI-related NeP (SCI-NeP)*	Central	103 (16.5)	Patients with 1) SCI (complete or incomplete paraplegia or tetraplegia) of at least 1 year duration with a nonprogressive (chronic) stage of at least 6 months duration and 2) NeP that started after the SCI and persisted continuously for at least 3 months or with remissions and relapses for at least 6 months.
Chronic low back pain with NeP (CLBP-NeP)	Peripheral	106 (17.0)	Subjects with low back pain persisting for at least 3 months with a confirmed NeP component based upon results from validated NeP screening tools.
Painful diabetic peripheral neuropathy (pDPN)	Peripheral	112 (17.9)	Patients with diabetic distal symmetrical sensory-motor polyneuropathy (peripheral neuropathy) with painful symptoms of at least 3 months duration.
Painful peripheral neuropathy with small fiber involvement (SFN)	Peripheral	100 (16.0)	Subjects diagnosed with painful peripheral neuropathy with small fiber involvement based upon history and physical exam, and either abnormal quantitative sensory testing findings or decrease in small fibers based on skin biopsy. Patients with small fiber neuropathy of known cause, including HIV, post-herpetic neuralgia, pDPN, or other hereditary forms of small fiber involvement should not be considered part of this NeP subtype.

* Subjects with SCI-related NeP who also have post-surgical pain were eligible to participate and considered to be in the SCI-NeP group.

HIV = human immunodeficiency virus; NeP = neuropathic pain; SCI = spinal cord injury; SFN = small fiber neuropathy.

**Table 2:** Subject characteristics, overall and by NeP severity level

Characteristic	Overall (N = 624)	Mild (N = 110)	Moderate (N = 297)	Severe (N = 207)	*P* value*
Age, years					0.0293
Mean (SD)	55.5 (13.74)	58.3 (15.10)	55.7 (13.19)	53.6 (13.33)	
Range	19–94	19–94	21–87	22–90	
Gender, N (%)					0.0222
Male	346 (55.4)	71 (64.5)	169 (56.9)	101 (48.8)	
Female	278 (44.6)	39 (35.5)	128 (43.1)	106 (51.2)	
Race, N (%)					0.0015
Missing	11 (1.8)	1 (0.9)	6 (2.0)	4 (1.9)	
American Indian or Alaska Native	9 (1.4)	1 (0.9)	3 (1.0)	5 (2.4)	
Asian	5 (0.8)	1 (0.9)	2 (0.7)	2 (1.0)	
Black or African American	100 (16.0)	13 (11.8)	37 (12.5)	47 (22.7)	
Native Hawaiian or other Pacific Islander	0 (0.0)	0 (0.0)	0 (0.0)	0 (0.0)	
White	448 (71.8)	89 (80.9)	230 (77.4)	122 (58.9)	
Multiracial	11 (1.8)	2 (1.8)	4 (1.3)	5 (2.4)	
Other	40 (6.4)	3 (2.7)	15 (5.1)	22 (10.6)	
Ethnicity, N (%)					0.0151
Missing	28 (4.5)	5 (4.5)	10 (3.4)	12 (5.8)	
Hispanic	53 (8.5)	5 (4.5)	21 (7.1)	27 (13.0)	
Not Hispanic	543 (87.0)	100 (90.9)	266 (89.6)	168 (81.2)	
Education level, N (%)					<0.0001
Missing	15 (2.4)	2 (1.8)	6 (2.0)	7 (3.4)	
Up to high school/GED	238 (38.1)	20 (18.2)	106 (35.7)	106 (51.2)	
Beyond high school	371 (59.5)	88 (80.0)	185 (62.3)	94 (45.4)	
Employment status, N (%)					<0.0001
Missing	12 (1.9)	4 (3.6)	3 (1.0)	5 (2.4)	
Employed for pay	118 (18.9)	30 (27.3)	64 (21.5)	24 (11.6)	
Disabled	294 (47.1)	27 (24.5)	131 (44.1)	129 (62.3)	
Retired	147 (23.6)	40 (36.4)	74 (24.9)	30 (14.5)	
Unemployed	36 (5.8)	6 (5.5)	16 (5.4)	14 (6.8)	
Other	17 (2.7)	3 (2.7)	9 (3.0)	5 (2.4)	
Time since first NeP symptoms, months					0.0330
Mean (SD)	113.9 (98.32)	93.7 (78.32)	115.4 (102.36)	121.5 (101.52)	
Range	0–725	6–393	0–725	5–603	
Time since NeP diagnosis, months					0.0059
Mean (SD)	93.9 (81.82)	75.9 (70.29)	94.8 (81.20)	101.7 (86.64)	
Range	6–592	6–393	6–534	6–592	
BPI-SF Pain Severity Index					N/A
N	614	110	297	207	
Mean (SD)	5.5 (2.21)	2.0 (1.09)	5.2 (0.80)	7.7 (1.05)	
Range	0–10	0–3	4–6	7–10	
Number of comorbid conditions†					<0.0001
Mean (SD)	3.2 (2.12)	2.5 (1.66)	3.0 (2.07)	3.8 (2.23)	
Range	1–11	1–11	1–9	1–9	

Note: Scores on the BPI Pain Severity Index were used to classify average pain severity. Ten subjects did not respond to all required items needed to calculate a BPI average pain severity score and thus were not included in any analysis by pain severity category (“missing”).

* *P* values are from the Kruskal–Wallis test for continuous variables; chi-square test for number of comorbid conditions; and Fisher's exact test for the remaining categorical variables; mild vs moderate vs severe.

† Among subjects with at least one comorbid condition.

BPI-SF = Brief Pain Inventory-Short Form; GED = General Education Development; NeP = neuropathic pain; N/A = not applicable; SD = ; standard deviation.

**Figure 1:**
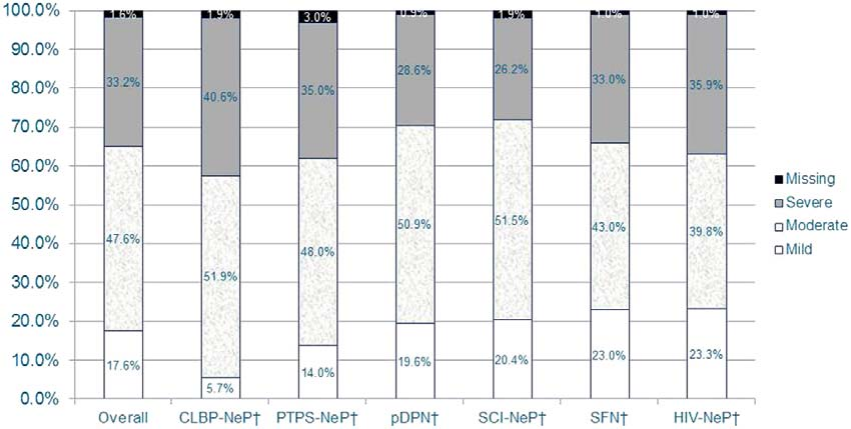
The majority of NeP subjects reported moderate or severe pain, regardless of NeP condition*.*Scores on the BPI-SF Pain Severity Index were used to classify average pain severity. Ten subjects did not respond to all required items needed to calculate a BPI-SF average pain severity score and thus were not included in any analysis by pain severity category (“missing”).^†^Pain severity levels for the individual NeP conditions have been previously published [15,20,37,38] or are being submitted for publication.BPI-SF = Brief Pain Inventory-Short Form; CLBP-NeP = chronic low back pain with a neuropathic pain component; HIV-NeP = human immunodeficiency virus-related peripheral neuropathic pain; NeP = neuropathic pain; pDPN = painful diabetic peripheral neuropathy; PTPS-NeP = post-trauma/post-surgery neuropathic pain; SCI-NeP = spinal cord injury-related neuropathic pain; SFN = painful peripheral neuropathy with small fiber involvement. [Color figure can be viewed in the online issue, which is available at http://wileyonlinelibrary.com.]

Subjects reported a mean (SD) pain severity score of 5.5 (2.2) overall. Close to half of the subjects (47.6%) reported moderate pain, while a third (33.2%) had severe pain. The majority of subjects reported suffering moderately to very strongly from a burning pain sensation (76.4%); a similar majority reported suffering moderately to very strongly from a prickling pain sensation (76.9%) (data not shown). The distribution of pain severity was similar across NeP conditions (Figure 1).

**Figure 2:**
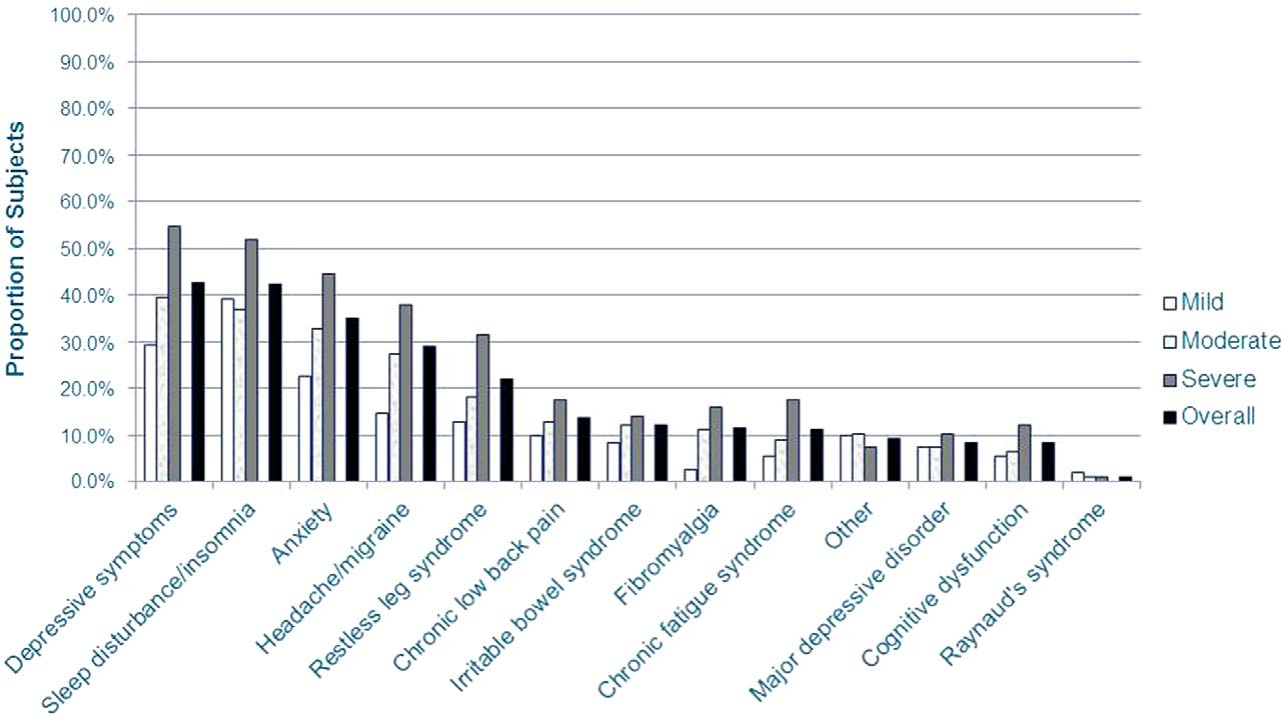
NeP subjects reported a variety of comorbid conditions*.*Scores on the BPI-SF Pain Severity Index were used to classify average pain severity. Ten subjects did not respond to all required items needed to calculate a BPI-SF average pain severity score and thus were not included in any analysis by pain severity category. A significant difference was observed across pain severity levels for depressive symptoms (*P* < 0.0001), sleep disturbance/insomnia (*P* = 0.0037), anxiety (*P* = 0.0003), headache/migraine (*P* < 0.0001), cognitive dysfunction (*P* = 0.0491), restless leg syndrome (*P* = 0.0001), chronic fatigue syndrome (*P* = 0.0019), and fibromyalgia (*P* = 0.0008).BPI-SF = Brief Pain Inventory-Short Form; NeP = neuropathic pain. [Color figure can be viewed in the online issue, which is available at http://wileyonlinelibrary.com.]

Overall, the mean (SD) time since NeP diagnosis was 7.8 (6.8) years, with a longer duration of NeP observed among those with greater pain severity (*P* = 0.0059). On average, the time from appearance of NeP symptoms to diagnosis was 20 months (1.7 years). Approximately three-fourths of the sample reported being originally diagnosed by a primary care physician (39.3%), a neurologist (23.9%), or a pain specialist (11.4%) (data not shown). On average, subjects had approximately 3 comorbidities, with more comorbidities among those with greater pain severity (*P* < 0.0001). The most common comorbidities overall were depressive symptoms (42.6%), sleep disturbance/insomnia (42.1%), and anxiety (35.1%) (Figure 2). Seventy-four (11.9%) subjects were not able to walk on their own; 50 of these subjects were SCI-NeP subjects.

Less than a fifth (18.9%) of the sample overall was employed for pay; the proportions of subjects who were disabled (47.1%) or who were retired (23.6%) were higher than the proportion employed for pay. A minority (12.2%) of subjects were receiving workers' compensation (data not shown). Finally, most subjects reported having some form of health insurance (93.2%), as well as NeP prescription drug coverage (87.3%; data not shown).

### Pain Interference with Function

The mean (SD) BPI-SF Pain Interference Index was 5.6 (2.5) overall, indicating moderate interference with function, and the mean Pain Interference Index score increased among those with greater pain severity (*P* < 0.0001) (Table 3). The standardized effect size (Cohen's *d*) for the Pain Interference Index when comparing mild and severe subjects was 2.68. Overall, the pain interference with function items most impacted (mean score above 5.0) by NeP were sleep, normal work, enjoyment of life, walking ability, and general activity (Table 3). A significant difference in mean scores was also observed across pain severity levels for each of the seven pain interference with function items (all *P* < 0.0001). The observed standardized effect size when comparing mild and severe subjects for all pain interference with function items was large, ranging from 1.63 to 2.83 (Table 3).

**Table 3:** Patient-reported pain interference with function, health status, and sleep, overall and by NeP severity level

Measure	Overall (N = 624)	Mild (N = 110)	Moderate (N = 297)	Severe (N = 207)	*P* value*	Cohen's *d*, Mild vs Severe†
BPI-SF pain interference with function‡						
Pain Interference Index					<0.0001	2.68
Mean (SD)	5.6 (2.51)	2.5 (2.01)	5.4 (1.94)	7.3 (1.69)		
Range	0–10	0–8	0–10	1–10		
General activity					<0.0001	2.83
Mean (SD)	5.6 (2.87)	2.1 (2.13)	5.5 (2.37)	7.6 (1.85)		
Range	0–10	0–10	0–10	1–10		
Mood					<0.0001	2.27
Mean (SD)	5.0 (3.04)	1.8 (2.04)	4.8 (2.65)	7.0 (2.39)		
Range	0–10	0–9	0–10	1–10		
Walking ability					<0.0001	1.97
Mean (SD)	5.8 (3.00)	2.8 (2.76)	5.6 (2.66)	7.5 (2.19)		
Range	0–10	0–10	0–10	1–10		
Normal work§					<0.0001	2.25
Mean (SD)	6.1 (2.89)	2.8 (2.67)	6.1 (2.47)	7.7 (1.93)		
Range	0–10	0–10	0–10	1–10		
Relations with other people					<0.0001	1.63
Mean (SD)	4.4 (3.11)	1.7 (2.29)	4.2 (2.77)	6.0 (2.85)		
Range	0–10	0–9	0–10	1–10		
Sleep					<0.0001	1.77
Mean (SD)	6.2 (3.15)	3.3 (3.09)	6.0 (2.82)	7.9 (2.36)		
Range	0–10	0–10	0–10	1–10		
Enjoyment of life					<0.0001	1.78
Mean (SD)	5.9 (3.03)	3.0 (2.76)	5.9 (2.67)	7.5 (2.43)		
Range	0–10	0–10	0–10	1–10		
SF-12v2¶						
Physical component summary					<0.0001	1.42
Mean (SD)	31.1 (9.55)	40.1 (10.64)	30.1 (8.35)	27.8 (7.49)		
Range	8–62	14–62	8–60	13–53		
Mental component summary					<0.0001	0.96
Mean (SD)	42.5 (12.41)	47.9 (11.95)	44.6 (11.68)	36.7 (11.49)		
Range	15–74	18–73	16–74	15–68		
Physical functioning					<0.0001	1.11
Mean (SD)	26.4 (29.79)	48.1 (34.64)	24.9 (28.01)	17.3 (23.72)		
Range	0–100	0–100	0–100	0–100		
Role physical					<0.0001	1.19
Mean (SD)	32.0 (26.93)	53.4 (32.43)	30.6 (23.70)	22.8 (21.57)		
Range	0–100	0–100	0–100	0–100		
Bodily pain					<0.0001	2.14
Mean (SD)	34.6 (27.42)	65.9 (25.95)	34.1 (22.48)	18.8 (19.74)		
Range	0–100	0–100	0–100	0–100		
General health					<0.0001	1.11
Mean (SD)	43.8 (26.62)	60.2 (22.99)	44.7 (26.15)	33.7 (24.53)		
Range	0–100	0–100	0–100	0–100		
Vitality					<0.0001	0.90
Mean (SD)	33.8 (26.42)	46.6 (26.34)	35.9 (24.91)	23.9 (24.92)		
Range	0–100	0–100	0–100	0–100		
Social functioning					<0.0001	1.17
Mean (SD)	48.5 (31.52)	67.3 (30.43)	52.2 (29.56)	33.7 (28.03)		
Range	0–100	0–100	0–100	0–100		
Role emotional					<0.0001	1.13
Mean (SD)	54.7 (31.58)	73.5 (30.09)	57.8 (29.83)	40.5 (28.69)		
Range	0–100	0–100	0–100	0–100		
Mental health					<0.0001	0.92
Mean (SD)	54.6 (23.56)	65.2 (22.61)	57.6 (21.82)	44.4 (22.83)		
Range	0–100	0–100	0–100	0–100		
MOS-SS**						
Sleep Problems Index					<0.0001	1.28
Mean (SD)	50.5 (20.10)	37.5 (18.31)	48.3 (18.41)	60.7 (18.26)		
Range	3–100	3–84	4–96	9–100		
Sleep disturbance					<0.0001	1.14
Mean (SD)	54.5 (27.44)	38.1 (25.56)	51.8 (25.43)	67.0 (25.48)		
Range	0–100	0–100	0–100	0–100		
Sleep adequacy					<0.0001	0.72
Mean (SD)	39.5 (24.81)	50.3 (24.99)	40.4 (23.53)	32.4 (24.83)		
Range	0–100	0–100	0–100	0–100		
Sleep somnolence					<0.0001	0.60
Mean (SD)	44.6 (24.69)	37.0 (23.94)	42.2 (22.93)	52.1 (25.75)		
Range	0–100	0–100	0–100	0–100		
Snoring					0.0166	0.31
Mean (SD)	40.2 (34.41)	32.3 (33.76)	41.1 (33.69)	42.9 (35.30)		
Range	0–100	0–100	0–100	0–100		
Shortness of breath or headache					<0.0001	0.86
Mean (SD)	23.0 (29.22)	8.2 (19.21)	21.4 (26.51)	33.2 (33.18)		
Range	0–100	0–100	0–100	0–100		
Sleep quantity					<0.0001	0.56
Mean (SD)	6.1 (1.92)	6.7 (1.74)	6.2 (1.71)	5.6 (2.17)		
Range	1–20	3–11	2–12	1–20		

Note: Scores on the BPI Pain Severity Index were used to classify average pain severity. Ten subjects did not respond to all required items needed to calculate a BPI average pain severity score and thus were not included in any analysis by pain severity category (“missing”).

* *P* values are from the Kruskal–Wallis test; mild vs moderate vs severe.

† An effect size of 0.2 to 0.3 may be considered a “small” effect, around 0.5 a “medium” effect, and 0.8 and higher may be considered a “large” effect [36].

‡ Lower scores indicate a better subject-reported outcome.

§ Includes work inside and outside the home.

¶ Higher scores indicate a better subject-reported outcome.

** Higher scores indicate more of the concept being measured. Higher scores for “sleep adequacy” and “sleep quantity” represent better sleep, whereas higher scores for the other scales indicate poorer sleep. BPI-SF = Brief Pain Inventory-Short Form; MOS-SS = Medical Outcomes Study Sleep Scale; SD = ; standard deviation; SF-12v2 = 12-item Short-Form Health Survey, version 2.

**Figure 3:**
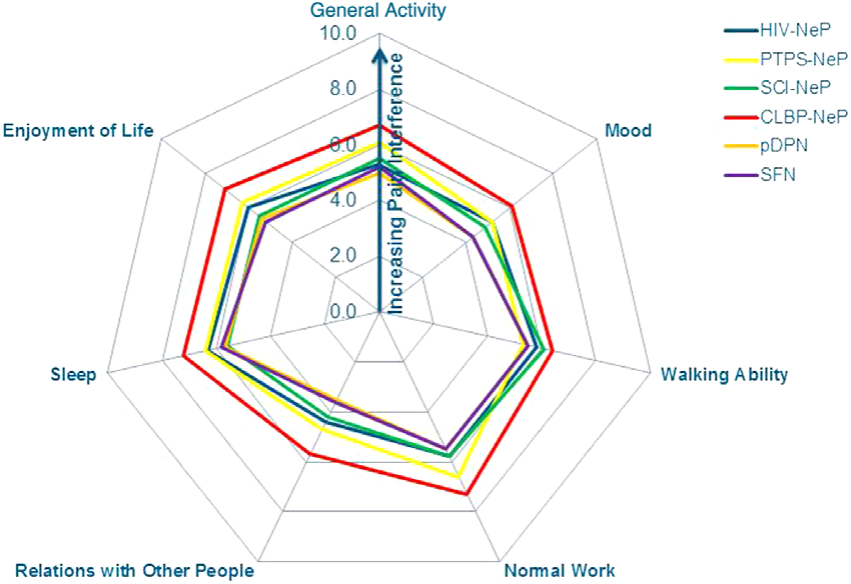
Pain interference with function trends is consistent across NeP conditions**Significant differences were observed across NeP conditions for all items (all *P* < 0.0010), except walking ability (*P* = 0.1612) and the Pain Interference Index (all *P* < 0.0010).CLBP-NeP = chronic low back pain with a neuropathic pain component; HIV-NeP = human immunodeficiency virus-related peripheral neuropathic pain; NeP = neuropathic pain; pDPN = painful diabetic peripheral neuropathy; PTPS-NeP = post-trauma/post-surgery neuropathic pain; SCI-NeP = spinal cord injury-related neuropathic pain; SFN = painful peripheral neuropathy with small fiber involvement. [Color figure can be viewed in the online issue, which is available at http://wileyonlinelibrary.com.]

The mean pain interference scores for each of the NeP conditions are presented in Figure 3. Mean scores exhibited a similar pattern across NeP conditions and were consistently the highest/worst among CLBP-NeP subjects.

### Health Status

The mean (SD) SF-12v2 PCS and MCS scores were 31.1 (9.6) and 42.5 (12.4) overall, respectively. Among subjects with more severe pain, PCS and MCS scores were lower/worse (both *P* < 0.0001); a large standardized effect size was observed for both when comparing mild and severe subjects: 1.42 and 0.96, respectively (Table 3). Average physical and mental health status overall and in each of the pain severity groups were lower/worse than the United States population norms (49.7 and 49.5, respectively) [28]. A significant difference was observed for each of the eight domains across pain severity levels (all *P* < 0.0001), and those with more severe pain had lower/worse mean scores on each of the eight domains (Table 3). The most negatively affected domain was physical functioning. When comparing mild and severe subjects, the observed standardized effect size for each of the eight SF-12 domains was large ranging from 0.90 to 2.14 (Table 3).

**Figure 4:**
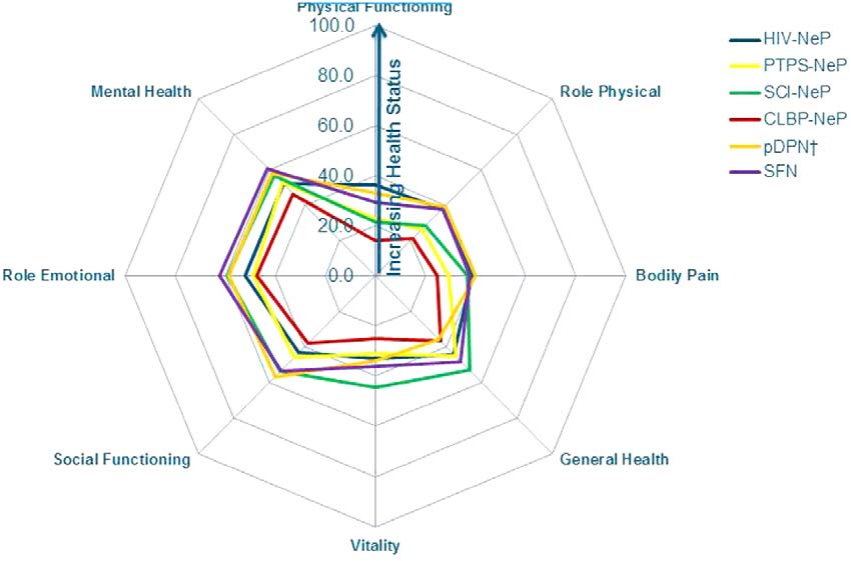
Trends across domains of physical and mental health are consistent across NeP conditions*.*Significant differences were observed across NeP conditions for all domains (all *P* < 0.003).^†^Mean summary scores for pDPN have been previously published [15].CLBP-NeP = chronic low back pain with a neuropathic pain component; HIV-NeP = human immunodeficiency virus-related peripheral neuropathic pain; NeP = neuropathic pain; pDPN = painful diabetic peripheral neuropathy; PTPS-NeP = post-trauma/post-surgery neuropathic pain; SCI-NeP = spinal cord injury-related neuropathic pain; SFN = painful peripheral neuropathy with small fiber involvement. [Color figure can be viewed in the online issue, which is available at http://wileyonlinelibrary.com.]

Mean SF-12v2 scores for each NeP condition are presented in Figure 4. Mean SF-12 scale scores exhibited a similar pattern across NeP conditions and were generally lowest/worst among CLBP-NeP subjects.

**Figure 5:**
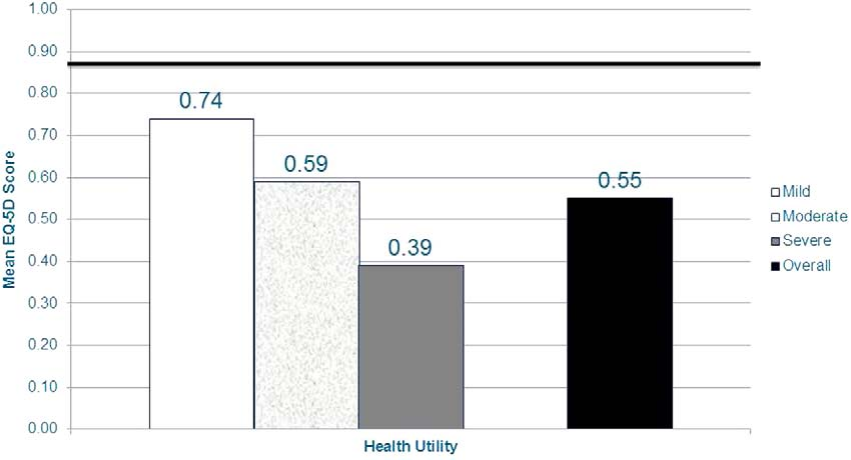
NeP subjects with more severe pain have worse general health status*.*Scores on the BPI-SF Pain Severity Index were used to classify average pain severity. Ten subjects did not respond to all required items needed to calculate a BPI-SF average pain severity score and thus were not included in any analysis by pain severity category. EQ-5D health state utility scored on a −0.11 to 1.00 scale; population norm (0.87 [39]) is indicated by the dark horizontal bar. A significant difference was observed across pain severity levels for EQ-5D health state utility (*P* < 0.0001). A large standardized effect size (Cohen's *d*) was observed for the EQ-5D health state utility when comparing mild and severe subjects: 2.03.BPI-SF = Brief Pain Inventory-Short Form; EQ-5D = EuroQol 5-dimensions; NeP = neuropathic pain. [Color figure can be viewed in the online issue, which is available at http://wileyonlinelibrary.com.]

The mean (SD) EQ-5D health utility was 0.55 (0.23) and decreased as pain severity increased (*P* < 0.0001) (Figure 5). The standardized effect size for the EQ-5D health utility when comparing mild and severe subjects was 2.03. Average scores overall and for each pain severity group were lower than the population norm of 0.87 [39].

### Sleep

The mean (SD) MOS-SS Sleep Problems Index was 50.5 (20.1) overall, which is markedly higher than the US population norm of 25.8 [30]. Among subjects with more severe pain, mean scores were higher/worse (*P* < 0.0001); a large standardized effect size (1.28) was observed for the Sleep Problems Index when comparing mild and severe subjects (Table 3). Those with more severe pain also had worse mean scores across all domains (all *P* < 0.02) (Table 3).

**Figure 6:**
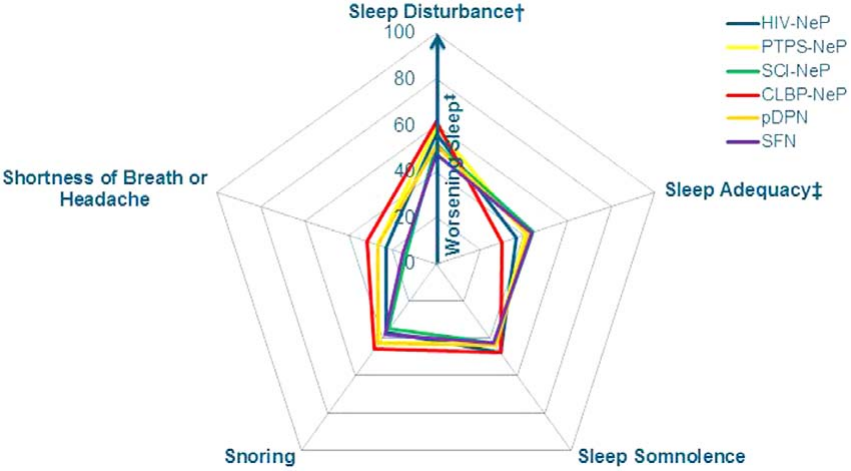
Trends across domains of sleep are consistent across NeP conditions*.*Significant differences were observed across NeP conditions for sleep disturbance, sleep adequacy, shortness of breath or headache domains (all *P* < 0.03).^†^Mean sleep disturbance for PTPS-NeP has been previously published [20].^‡^The sleep adequacy item differs from all others in that higher scores indicate better outcomes on this item.CLBP-NeP = chronic low back pain with a neuropathic pain component; HIV-NeP = human immunodeficiency virus-related peripheral neuropathic pain; NeP = neuropathic pain; pDPN = painful diabetic peripheral neuropathy; PTPS-NeP = post-trauma/post-surgery neuropathic pain; SCI-NeP = spinal cord injury-related neuropathic pain; SFN = painful peripheral neuropathy with small fiber involvement. [Color figure can be viewed in the online issue, which is available at http://wileyonlinelibrary.com.]

The mean MOS-SS subscale scores for each of the NeP conditions are presented in Figure 6. Mean scores exhibited a similar pattern across NeP conditions and were consistently the worst among CLBP-NeP subjects.

### Anxiety and Depression

**Figure 7:**
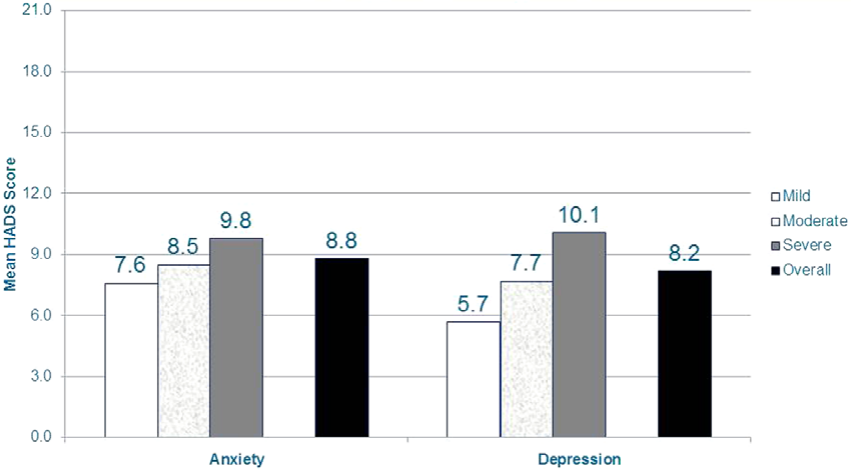
NeP subjects with more severe pain have more anxiety and depression*.*Scores on the BPI-SF Pain Severity were used to classify average pain severity. Ten subjects did not respond to all required items needed to calculate a BPI-SF average pain severity score and thus were not included in any analysis by pain severity category. HADS anxiety and depression scales scored on a 0–21 scale, where 0–7 is normal, 8–10 is mild, 11–14 is moderate, and 15–21 is severe. Significant differences were observed across pain severity levels for the anxiety and depression scales (both *P* < 0.0001). A medium standardized effect size (Cohen's *d*) was observed for the anxiety scale when comparing mild and severe subjects: 0.59. A large standardized effect size (Cohen's *d*) was observed for the depression scale when comparing mild and severe subjects: 1.02.BPI-SF = Brief Pain Inventory-Short Form; HADS = Hospital Anxiety and Depression Scale; NeP = neuropathic pain. [Color figure can be viewed in the online issue, which is available at http://wileyonlinelibrary.com.]

The majority of subjects have some level of anxiety (61.9%) and depression (54.3%). The mean (SD) HADS anxiety and depression scores were 8.8 (3.6) and 8.2 (4.5) overall, respectively; indicating mild levels of anxiety and depression. Similar levels were seen across each of the NeP conditions. Among subjects with more severe pain, mean scores were higher/worse (both *P* < 0.0001), and the standardized effect size when comparing mild and severe subjects was 0.59 for anxiety and 1.02 for depression (Figure 7).

## Discussion

To our knowledge, this is the first study in the United States to comprehensively evaluate the patient-reported burden of illness associated with broad NeP, that is, both peripheral and central NeP. Overall, subjects in this study reported a substantial degree of impairment; NeP subjects in the sample experienced substantially worse health status and sleep than the general US population norms [30],[28],[39].

The suboptimal patient-reported outcomes we observed were consistent with previous studies in NeP samples 7–12,14,21–26. For example, in previous studies assessing health status in NeP, subjects reported similar mean health utilities, ranging from 0.44 (using EQ-5D) [7] to 0.56 (using SF-6D) [24] in broad European NeP samples and 0.50 (using EQ-5D) [11],[12] in a American pDPN sample. Comparable physical and mental health status scores measured using the SF-12 PCS and MCS have also been reported among broad European NeP samples: PCS ranging from 31.29 [24] to 38.9 [23] and MCS ranging from 40.2 [24] to 40.8 [23]. NeP subjects from a broad sample in France reported a higher degree of sleep impairment (mean MOS-SS Sleep Problems Index 47.4) compared with subjects in our study [23]. Anxiety and depression scores measured using the HADS in our study were comparable with scores among pDPN subjects in Asia, Latin America, and the Middle East (mean anxiety and depression 8.9 and 7.9, respectively) [10].

Subjects in this study were required to have been diagnosed with NeP at least 6 months prior and were actively seeking care. Despite this, the majority of subjects reported a moderate or severe level of pain, which is consistent with findings previously reported [7],[11],[14].

In addition, we observed an association between pain severity and patient-reported outcomes, including health status, physical and mental health, pain interference with function, sleep, and mood; severe pain subjects were negatively impacted by 30% or more for each of these measures compared with mild pain subjects. The observed standardized effect size (Cohen's *d*) on these scales ranged from 0.59 for anxiety to 2.68 for pain interference with function, and a large effect size (>0.95) was observed for all scales, except anxiety, when comparing mild and severe subjects. In addition, when comparing mild and severe subjects, we observed a medium or large effect size for all individual domains, except the snoring domain of the MOS-SS (0.31). We found subjects with severe pain had the highest levels of pain interference with function (mean 7.3), consistent with a broad NeP burden of illness assessment conducted in France, Germany, Italy, the Netherlands, Spain, and United Kingdom (mean 6.8) [7]. Subjects with severe pain in our sample also had the worst health status (mean SF-12v2 PCS and MCS 27.8 and 36.7, respectively, and mean EQ-5D 0.39); in other studies assessing health status in NeP, subjects with severe pain have reported similar mean EQ-5D health utilities: 0.16 (broad NeP sample in Europe) [7], 0.2 (American pDPN sample) [11], and 0.27–0.36 (pDPN samples in Asia, Latin America, and Middle East) [10]. Finally, subjects in our sample with severe pain reported marked sleep problems (mean MOS-SS Sleep Problems Index 60.7), which is consistent with previous studies [10],[11],[15].

Based on a variety of patient-reported outcome measures, the findings of this study suggest that the humanistic burden of NeP is notable. While it is interesting to understand which NeP patient(s) experience the greatest burden, we observed similarities in function, health status, and sleep across the 6 NeP conditions (Figures 3, 4, and 6). Our findings were most prominent in CLBP-NeP subjects, which is an important finding given the high (up to 55%) prevalence of NeP among individuals with CLBP in the US population [6,40,41]. Finally, across NeP conditions, a consistent driver of humanistic burden was high pain severity.

Despite most patients taking medications to treat their NeP, the subjects in our study reported high levels of pain and other symptoms, including depression, anxiety, and sleep disturbance. This suggests that the management of NeP remains a difficult challenge for patients and their health care providers. These findings also support published guidelines [42], where tailored NeP management strategies are advised to include evaluations of depression, anxiety, sleep, pain, and interference with function.

### Limitations

We wish to acknowledge several limitations, particularly related to the possibility of incurring selection bias. Enrolled subjects were actively seeking medical care for their NeP; subjects had been managed at the study site for at least 6 months prior to enrollment and were approached to participate in the study as they presented for a routine medical appointment. As a result, the proportion of NeP subjects in our study with moderate or severe pain may be different than among all NeP patients. For example, active patient management may lead to symptom improvement. Therefore, while our sample represents NeP patients in routine clinical practice, findings may not be generalizable to others with NeP who are not seeking treatment or do not regularly visit their physician.

Furthermore, this study did not evaluate patient's treatment compliance or whether prescribed medications were always filled. Thus, it is possible that the observed proportion of subjects with moderate or severe pain is attributable, in part, to lack of compliance with treatment.

While inclusion criteria required that subjects were diagnosed with NeP at least 6 months prior to enrollment, there is a possibility that a proportion of subjects in the study were misdiagnosed. On average, subjects had been diagnosed with NeP more than 7 years prior to enrollment, with over one-third diagnosed by a neurologist or pain specialist. Clinical characteristics of the sample are consistent with NeP, for example, a majority of subjects reported suffering from burning and prickling pain sensations, and with previously published research. Nevertheless, we cannot be sure of the extent of the impact of misdiagnosis on our results. Finally, subjects who chose to enroll in the study and complete the questionnaires may have reported more improved scores with respect to pain, sleep, function, etc., compared with patients who did not receive this additional attention from their health care providers (i.e., Hawthorne effect).

Pain and function were assessed at one time point in our study, while individuals with NeP may experience day-to-day fluctuations in pain and function. We report average results in pain and function across a large sample of 624 subjects to describe the average impact of NeP; however, results for each subject may not capture that subject's typical experience.

Additionally, the BPI-SF Pain Severity Index was used to classify subjects as mild, moderate, or severe [43]. These cutoff scores have previously been validated using a sample of patients with pDPN and may not be the appropriate cutoff points for other types of NeP. However, Serlin and colleagues identified similar cutoffs (mild [1 to 4], moderate [5 to 6], and severe [7 to 10]) for assessing pain severity among cancer patients [44].

The use of generic, rather than disease-specific instruments to evaluate pain and other patient-reported outcomes, could also be a study limitation [45],[46]. Using these generic measures, it may have been difficult for subjects to distinguish between NeP and other pain. However, in previous NeP research, these measures have been used to demonstrate differences between groups and across time. Future research among patients with NeP and controls without NeP could be useful to better understand the incremental burden of NeP compared with the underlying condition.

### Summary

Subjects across NeP conditions exhibited high pain levels. Pain severity was statistically significantly associated with poor function, compromised health status and sleep, and increased anxiety and depression. Of the 6 NeP conditions, the observed burden was most substantial among CLBP-NeP, although the pattern of NeP impact was similar regardless of NeP condition. Results of this cross-sectional study indicate substantial patient burden across broad NeP, particularly among subjects with severe pain.
